# Draft Genome Sequences of Ciliovirus and Brinovirus from San Francisco Wastewater

**DOI:** 10.1128/genomeA.00651-15

**Published:** 2015-06-25

**Authors:** Alexander L. Greninger, Joseph L. DeRisi

**Affiliations:** a Department of Biochemistry and Biophysics, UCSF, San Francisco, California, USA; b Howard Hughes Medical Institute, UCSF, San Francisco, California, USA

## Abstract

We report the draft genome sequences of ciliovirus and brinovirus, two members of a likely new family of RNA viruses assembled from San Francisco wastewater. Based on sequence alignments and a nonuniversal genetic code, we believe these to be the first described RNA viruses of ciliates; however, more work is necessary to confirm their host.

## GENOME ANNOUNCEMENT

Ciliates are large single-cellular protozoal organisms defined by the presence of cilia as well as macro- and micronucleus. Ciliates are also noted for possessing a nonuniversal genetic code. Virus-like particles have been reported to be associated with *Hyalophysa chattoni*, and a large DNA virus, chlorella virus, has been isolated from *Paramecium bursaria* (1, 2). However, no RNA virus has been recovered from ciliates to date (3).

While performing weekly metagenomic sequencing of San Francisco wastewater, we recovered two contigs of 10,381 and 9,565 nucleotides that distantly aligned by BLASTx to a small portion of an RNA-dependent RNA polymerase of various members of the *Flaviviridae*. Translation of the two contigs using the universal genetic code failed to reveal open-reading frames (ORFs) of >500 nucleotides. However, translation of two contigs utilizing the ciliate genetic code revealed ORFs of 9,954 and >9,260 nucleotides that preserved the BLASTx alignments. HHPred analysis of the contigs translated in the ciliate genetic code revealed a superfamily II helicase similar to eIF4A followed by a picornavirus/flavivirus-like RNA-dependent RNA polymerase followed by a birnavirus/sobemovirus-like viral capsid protein, suggesting a genome organization most similar to that of the *Potyviridae* (3, 4). Of note, the two polyproteins were 28.7% identical by amino acid to each other throughout the ORF and 40% identical by amino acid to each other in the putative RNA-dependent RNA polymerase and helicase regions, consistent with these two contigs forming two new genera of a new viral family.

Notably, both contigs were recovered in only one wastewater sample from 25 January 2010 that was taken after a large rainstorm that left >5 inches of rain over the preceding week. Over one-quarter of the alignable nonchordate eukaryotic reads from the DNAsed sample aligned to *Ciliophora* organisms with half of these reads belonging to the *Tetrahymenidae* family, making it one of the most abundant organisms in the sample and strengthening the case for these viruses having ciliate hosts (5).

Sample processing was performed on 1 liter of wastewater that was concentrated to <5 mL with particles between the sizes of 0.22 μm and 300 kDa using Millipore Pellicon XL 300-kDa filters and 0.22-μm spin columns. Viral particle-enriched sample was treated with micrococcal nuclease and nucleic acid was extracted using a Zymo Viral DNA/RNA kit and half of the recovered nucleic acid was treated with DNase. Both contigs were assembled using PRICE v1.0 and Geneious v8.0 Assembler with metagenomic analysis using SURPI v1.0 from a total of 15,719,690 paired-end 65-bp reads sequenced on an Illumina GAIIx split between these DNAsed and untreated nucleic acid preparations (5, 6). Average coverage of the two contigs was 550× and 1271×, respectively. Coverage of ciliovirus in the DNAsed sample was 3.5× higher than in the untreated sample, while coverage of brinovirus was 2.4× higher in the DNase-treated sample, consistent with these being RNA viruses.

### Nucleotide sequence accession numbers.

The GenBank accession numbers for ciliovirus and brinovirus are JN661159 and KF412899, respectively.
